# Unveiling the microbiota-metabolite-myocardium axis: a novel perspective on cardiovascular health

**DOI:** 10.3389/fmicb.2024.1389311

**Published:** 2024-05-09

**Authors:** Zhenhua Guo, Yangfang Zhong, Le Zhou, Peier Xu, Naijing Gao, Jinyue Lu, Xueyun Yan, Huaming Cao

**Affiliations:** ^1^Department of Cardiology, Shibei Hospital, Shanghai, China; ^2^Shanghai Jing’an District Pengpu Town Second Community Health Service Center, Shanghai, China

**Keywords:** myocardial infarction, microbiota-metabolite-myocardium axis, 16S rDNA, metabolomics, LC-MS

## Abstract

**Introduction:**

Cardiovascular diseases, including myocardial infarction, remain a leading cause of death globally. Emerging evidence suggests the gut microbiota plays a crucial role in cardiovascular health. This study aims to explore the impact of gut microbiota on myocardial infarction using a mouse model.

**Methods:**

The research utilizes a multi-omics approach, including 16S rDNA sequencing and LC-MS-based metabolomics to analyze fecal and serum samples from mice modeled to mimic myocardial infarction. This methodology allows for a comprehensive analysis of microbial populations and their metabolic output.

**Results:**

The findings reveal a significant reduction in gut microbiota α-diversity in mice with induced myocardial infarction compared to healthy controls. Notably, there is an increase in populations of Fusobacteria and Clostridia. Metabolomic analysis indicates disruptions in amino acid and energy metabolism, suggesting a metabolic dysregulation linked to myocardial health.

**Discussion:**

The study proposes a novel microbiota-metabolite-myocardium axis, where specific microbial metabolites may directly affect heart health. This connection points to the gut microbiota as a potential player in the pathogenesis of myocardial infarction and may open new therapeutic avenues targeting the gut microbiome to combat cardiovascular diseases.

## 1 Introduction

Myocardial infarction, a critical issue within cardiovascular health, emerges due to insufficient coronary arterial blood flow, leading to myocardial necrosis (Smit et al., 2020). This condition presents through various clinical symptoms, notably chest pain, ECG changes, and elevated cardiac enzymes (Gong et al., 2020). Myocardial infarction is classified into two primary types: ST-segment elevation myocardial infarction (STEMI) and non-ST-segment elevation myocardial infarction (NSTEMI), with NSTEMI being more subtle and less pronounced in its clinical manifestations (Byrne et al., 2024). The consequences of myocardial infarction are severe, potentially resulting in cardiogenic shock, arrhythmias, and significantly affecting patient quality of life, highlighting the critical nature of this condition (Jung et al., 2021; Samsky et al., 2021; Sterling et al., 2023). The incidence of myocardial infarction has been on the rise, with China reporting approximately 3 million new cases annually (Song et al., 2020). Despite advancements in medical and interventional treatments, the short-term mortality rate for myocardial infarction patients can reach up to 7.5%, and the five-year survival rate does not exceed 50–60% (Song et al., 2020). These statistics underscore myocardial infarction as a leading cause of cardiovascular mortality (Yang et al., 2022). Current treatment strategies, such as thrombolysis and recanalization, are vital yet insufficient to reverse ischemic injury (Widimsky et al., 2023). This underscores the necessity for a deeper understanding of myocardial infarction’s pathogenesis and the exploration of new therapeutic targets, emphasizing the ongoing quest for more effective treatments in the battle against this life-threatening condition.

One of the pivotal etiological factors of myocardial infarction is identified as coronary atherosclerosis, which precipitates thrombus formation leading to occlusion (Ambrose and McEnroe, 2022). The progression towards this occlusive thrombus is not linear but is facilitated by a cascade of physiological disruptions including inflammatory responses, oxidative stress, endothelial dysfunction, among others (Carresi et al., 2021). Beyond these, metabolic disorders have been recognized as significant contributors to the risk and development of acute myocardial infarction (Hasebe and Hasebe, 2022). Perturbations in energy metabolism, particularly within the tricarboxylic acid cycle, have been documented in the context of myocardial infarction, suggesting a critical impairment in energy substrate utilization during cardiac injury (Sanchez-Gimenez et al., 2023). Intriguingly, recent explorations have begun to unravel a potential linkage between myocardial infarction and gut microbiota dysbiosis, although the specific mechanistic pathways remain to be fully elucidated. This nascent area of research opens a novel vista for understanding myocardial infarction, positing the gut microbiome as a significant player in the modulation of cardiovascular health. The interplay of traditional factors such as coronary atherosclerosis and metabolic disorders with emerging insights into microbiota dysbiosis underscores the multifaceted nature of myocardial infarction pathogenesis, heralding a new era of comprehensive cardiovascular research and therapeutic strategies.

The human gut is a vast ecosystem, teeming with trillions of microbes, collectively termed the gut microbiome (Gomaa, 2020). The advent of advanced genomic sequencing technologies has facilitated a profound understanding of these microbial communities and the genetic material they harbor (Rezasoltani et al., 2020). This groundbreaking research has established the gut microbiome as a fundamental pillar of human physiology, exerting considerable influence over metabolism, immune function, and nutritional status (Wiertsema et al., 2021). Dysbiosis, a term denoting the disruption of this microbial equilibrium, has been implicated in a myriad of health conditions, notably type 2 diabetes and cardiovascular diseases (Tian et al., 2021). The modulation of the gut microbiome emerges as a promising avenue for the prevention and treatment of cardiometabolic disorders.

Recent investigations have highlighted alterations in the gut microbiota composition of patients with myocardial infarction, including an enhanced prevalence of Fusobacteria (Mutalub et al., 2022). These findings, albeit preliminary, underscore the potential interplay between gut microbiota and cardiovascular health. However, the exploration of this relationship is currently hampered by limitations such as small study cohorts and the reliance on singular methodological approaches. Comprehensive analyses focusing on the gut microbiota’s role in myocardial infarction are imperative for identifying pivotal pathogenic factors and novel therapeutic targets. Short-chain fatty acids (SCFAs), including acetate, propionate, and butyrate, are metabolites produced by gut microbes, such as those from the Lachnospiraceae and Ruminococcaceae families, through the fermentation of dietary carbohydrates (Song et al., 2021). These SCFAs are integral not only for the energy supply of colonocytes and the maintenance of intestinal barrier integrity but also for their systemic effects on cholesterol and lipid metabolism via receptor interactions (He et al., 2020).While prior research has concentrated on these and other gut microbiota-derived metabolites, such as TMAO and carnitine, offering insights into the pathogenesis of heart attacks, a holistic approach to study the disease in its entirety has been lacking (Wang et al., 2022).

The objective of this study is to develop a mouse model of myocardial infarction to methodically examine the variances in gut microbiota structure and metabolic profiles between afflicted and control groups. Through the integration of multi-omics data, this research aims to delineate the pathogenesis network of myocardial infarction, thereby providing a scientific foundation for the development of targeted therapeutic interventions. This methodology is poised to offer a comprehensive overview of the microbiota-cardiovascular axis, elucidating the connections between microbial metabolites and crucial host pathways and target genes. This endeavor is anticipated to facilitate personalized risk assessment and intervention strategies for cardiovascular diseases, leveraging a systems biology approach to unveil new mechanistic insights into myocardial infarction.

## 2 Methods

### 2.1 Experimental animals

Twenty healthy male C57BL/6 mice (SPF grade) weighing 20 ± 2 g at 8 weeks old were purchased from Shanghai SLAC Laboratory Animal Co., Ltd. After transport to the lab, they were housed in a specific pathogen-free environment at 22 ± 2°C and 60 ± 5% humidity with free access to food and water and a 12 h light/dark cycle. After acclimatization for 1 week, the mice were randomly divided into control and model groups (*n* = 10 each) using a random number table. Strict national experimental animal management regulations were followed to provide suitable breeding conditions. All animal experiments were approved by the ethics committee of Shibei Hospital. To induce the model of MI, occlusion of the left anterior descending coronary artery (LAD) was employed. In brief, mice were anaesthetized with R520 mobile small animal anesthesia machine (Reward Life Technology Co., Ltd., Shenzhen, China). The skin, fat and muscle were gently separated and then the fourth intercostal space on the left side of the chest was exposed. Left thoracotomy was performed to expose the heart, and the left anterior descending (LAD) coronary artery was visualized. An 8-0 nylon suture was passed under the LAD and ligated 1–2 mm below the tip of the left atrium. Occlusion was confirmed by observing blanching of the anterior wall of left ventricle post ligation. The lungs were inflated by increasing positive pressure ventilation before closing of thoracic cavity. Analgesics were administered post-surgery for pain management. Animal experiments were conducted after approval by the Ethics Committee of the Shanghai Shibei Hospital, and the ethical approval number is: SHIB-2022191.

After surgery, the mice were randomly divided into two groups (*n* = 10): (A) sham group (without LAD), (B) MI group (with LAD). The serum samples were collected for serum untargeted metabolomics analysis. All rats were sacrificed under the deeply anesthetized condition after echocardiographic examinations. The serum fecal and samples were collected for 16 s rDNA sequencing.

### 2.2 Assessment of cardiac function and histological analysis

Two weeks after the induction of MI, all mice were anaesthetized lightly with 1.5% isoflurane and mice undergo echocardiographic assessment (Vevo 2100) to evaluate cardiac function. Measurements include left ventricular ejection fraction (LVEF), left ventricular fractional shortening (LVFS). These parameters are used to assess the extent of cardiac dysfunction and remodeling following MI.

After echocardiographic assessment, mice are euthanized, and hearts are excised and fixed in formalin. Paraffin-embedded heart sections are stained with Masson’s trichrome to quantify fibrosis, with collagen fibers appearing blue and cardiac muscle fibers appearing red. Hematoxylin and eosin (H&E) staining is performed to evaluate general tissue morphology, including inflammation and necrosis. Terminal deoxynucleotidyl transferase dUTP nick end labeling (TUNEL) staining is used to detect apoptotic cells, with positive cells appearing as dark brown or black nuclei.

### 2.3 Metabolomics experiment

In the metabolomics study, 100 μL of serum samples were initially combined with 300 μL of chilled methanol, which included phenylalanine as an internal standard. This mixture was then vortexed for 3 min and subsequently centrifuged at 12,000 rpm for 10 min at a temperature of 4°C. The resulting supernatant was carefully transferred into autosampler vials, prepared for LC-MS/MS analysis.

The analysis of the metabolic profile was conducted using a Dionex Ultimate 3000 RS UHPLC system, coupled with a Q-Exactive Plus quadrupole-Orbitrap mass spectrometer. This system was equipped with a heated electrospray ionization (ESI) source from Thermo Fisher Scientific, Waltham, MA, United States, and was operated in both positive and negative ESI ion modes. For the chromatographic separation, an ACQUITY UPLC HSS T3 column (1.8 μm, 2.1 × 100 mm) was utilized in both ionization modes.

The separation was accomplished using a binary gradient elution system, comprising of solvent A (water with 0.1% formic acid, v/v) and solvent B (acetonitrile with 0.1% formic acid, v/v). The gradient program was set as follows: starting with 5% B at 0 min, maintaining 5% B till 2 min, increasing to 25% B at 4 min, 50% B at 8 min, 80% B at 10 min, reaching 100% B at 14 min and holding till 15 min, then returning to 5% B at 15.1 min and holding till 16 min. The flow rate was maintained at 0.35 mL/min, and the column temperature was controlled at 45°C. All samples were kept at a constant temperature of 4°C during the analysis. The injection volume for each sample was 2 μL, and the mass spectrometer was set to scan a mass range from *m*/*z* 100 to 1,000.

The resolution settings for the mass spectrometer were 70,000 for full MS scans and 17,500 for HCD MS/MS scans. The collision energies were adjusted to 10, 20, and 40 eV. The operational parameters for the mass spectrometer included a spray voltage of 3,800 V in the positive mode and 3,000 V in the negative mode, a sheath gas flow rate of 35 arbitrary units, an auxiliary gas flow rate of 8 arbitrary units, a capillary temperature of 320°C, an auxiliary gas heater temperature of 350°C, and an S-lens RF level set at 50. Quality control samples (QCs) were injected at regular intervals throughout the analytical run, providing a dataset to evaluate the repeatability of the analysis.

### 2.4 Data preprocessing and statistical analysis for metabolomics

The LC–MS data were processed using Progenesis QI V2.3 software (Nonlinear Dynamics, Newcastle, United Kingdom). This processing included several critical steps: baseline filtering, peak identification, integration, retention time correction, peak alignment, and normalization. Key settings for this process were a precursor tolerance of 5 ppm, product tolerance of 10 ppm, and a product ion threshold of 5%.

Compound identification hinged on the accurate mass-to-charge ratio (*m*/*z*), MS1/MS2 fragment ions, and isotopic distribution. This identification utilized several databases, including The Human Metabolome Database (HMDB), Lipid Maps (V2.3), Metlin, EMDB, and PMDB. The structures of the compounds were further elucidated using our library of internal standard metabolites, ensuring a match in exact masses, fragment ion masses, and retention times.

The data extracted underwent additional processing. Peaks with missing values (ion intensity = 0) in more than half of the samples within any group were removed. Zero values were substituted with half of the minimum observed value. Screening was also conducted based on the qualitative results of the compounds, discarding those with scores below 36 out of 60, as they were considered inaccurate.

A comprehensive data matrix was then compiled from both positive and negative ion data sets. This matrix was imported into R software for Principal Component Analysis (PCA), which helped in assessing the overall distribution of the samples and the stability of the analytical process.

For more detailed analysis, Orthogonal Partial Least-Squares-Discriminant Analysis (OPLS-DA) were employed. These methods were used to differentiate metabolites across various groups. To ensure the robustness of these models and prevent overfitting, a 7-fold cross-validation and 200 Response Permutation Testing (RPT) were performed.

Variable Importance of Projection (VIP) scores from the OPLS-DA model were utilized to determine the significance of each variable in group discrimination. A two-tailed student’s *t*-test was then applied to ascertain the statistical significance of the differences in metabolites between groups. Differential metabolites were ultimately selected based on VIP values exceeding 1.0 and *p*-values lower than 0.05.

### 2.5 Microbiological experiment

The extraction of DNA was carried out with the DNeasy PowerSoil Kit, strictly following the protocol provided by the manufacturer. The extracted DNA’s concentration was determined using the Quant-IT PicoGreen kit from Invitrogen. For the preparation of the sequencing library, protocols from the Illumina 16S Metagenomic Sequencing Library were utilized, specifically targeting the amplification of the V3 and V4 regions. A total of 2 ng of genomic DNA (gDNA) was amplified through PCR, employing a composition of 1× reaction buffer, 1 nM dNTP mix, 500 nM of each universal F/R PCR primer, and 2.5 U of Herculase II fusion DNA polymerase provided by Agilent Technologies, Santa Clara, CA, United States. The PCR cycling conditions were set as follows: an initial heat activation for 3 min at 95°C, followed by 25 cycles each consisting of 30 s at 95°C, 30 s at 55°C, and 30 s at 72°C, concluding with a final extension phase of 5 min at 72°C. The primer pair used for this initial amplification included universal primers with Illumina adapter overhang sequences, specifically V3-F: 5′-TACGGRAGGCAGCAG-3′ and V4-R: 5′-AGGGTATCTAATCCT-3′. This initial PCR product was then purified using AMPure beads from Agencourt Bioscience, Beverly, MA, United States. Post-purification, 10 μL of this product underwent further PCR amplification using the NexteraXT Indexed Primer for the final assembly of the library, following the same cycling conditions as the initial PCR, but limited to 10 cycles. The resultant PCR product was again purified using AMPure beads. This final purified product was quantified via qPCR, in line with the qPCR Quantification Protocol Guide from the KAPA Library Quantification kits designed for Illumina Sequencing Platforms, and its quality was assessed using a TapeStation D1000 ScreenTape from Agilent Technologies, Waldbronn, Germany. The final step involved paired-end sequencing (2 × 300 bp) performed by the MiSeq^™^ platform from Illumina.

### 2.6 16S rDNA data processing

Raw paired-end 16S rDNA sequences from the V3–V4 region were consolidated into consensus fragments using FLASH software (version 1.2.11). This step was followed by a filtration process where reads were clustered at 97% similarity with the aid of CD-HIT-OTU. This process involved the detection and removal of chimeric sequences and discarding reads of low quality, ambiguous nature, or those shorter than 400 bp. The high-quality reads that remained were then grouped into operational taxonomic units (OTUs) using a greedy algorithm.

Taxonomic categorization of these OTUs was carried out using the BLAST tool (version 2.4.0). Subsequent microbial community analyses, including evaluations of alpha-diversity and beta-diversity, as well as principal coordinate analyses (PCoA), were conducted utilizing the QIIME software (version 1.0). Alpha-diversity metrics such as Chao1, Shannon, and inverse Simpson indices were calculated. Beta-diversity, on the other hand, was assessed through PCoA based on weighted UniFrac distances.

The normality of data distribution was tested using Shapiro–Wilk’s test. Differences in relative abundance of microbial communities were examined using statistical approaches like Student’s t-test or Wilcoxon rank sum test, depending on the data suitability. To adjust for the risk of false findings due to multiple hypothesis testing, the false discovery rate (FDR) method was employed.

For the identification of potential metagenomic biomarkers, Linear Discriminant Analysis Effect Size (LEfSe) was utilized. This technique involves class comparison, checks for biological consistency, and estimates the size of the effects. Visual representation of differentially abundant functional categories, with an LDA score of 2.0 or higher, was achieved through heatmap and PCoA analyses.

Additionally, the Analysis of Similarities (ANOSIM) was used to compare matrices of rank dissimilarities. For functional gene enrichment analysis, the PICRUSt (Phylogenetic Investigation of Communities by Reconstruction of Unobserved States) approach was employed. This method estimates the proportional contributions of each sample’s KEGG categories, aiding in the understanding of functional aspects of the microbial communities.

### 2.7 Multi-omics data integration analysis

To trace the origin of metabolites through database search, researchers conduct Metabolic Pathway Enrichment Analysis (MPEA) based on different sources. By utilizing the powerful Sankey network, they integrate and visualize the correlations at both biological and statistical levels. MetOrigin^1^ was first utilized to conduct Spearman correlation analysis between gut microbiota and metabolomics. MetOrigin is a bioinformatics tool, aiming to identify which bacteria and how they participate in certain metabolic reactions, helping us to understand where metabolites come from: host, bacteria, or both.

## 3 Result

### 3.1 Echocardiographic and histological observations

**Figure 1:**
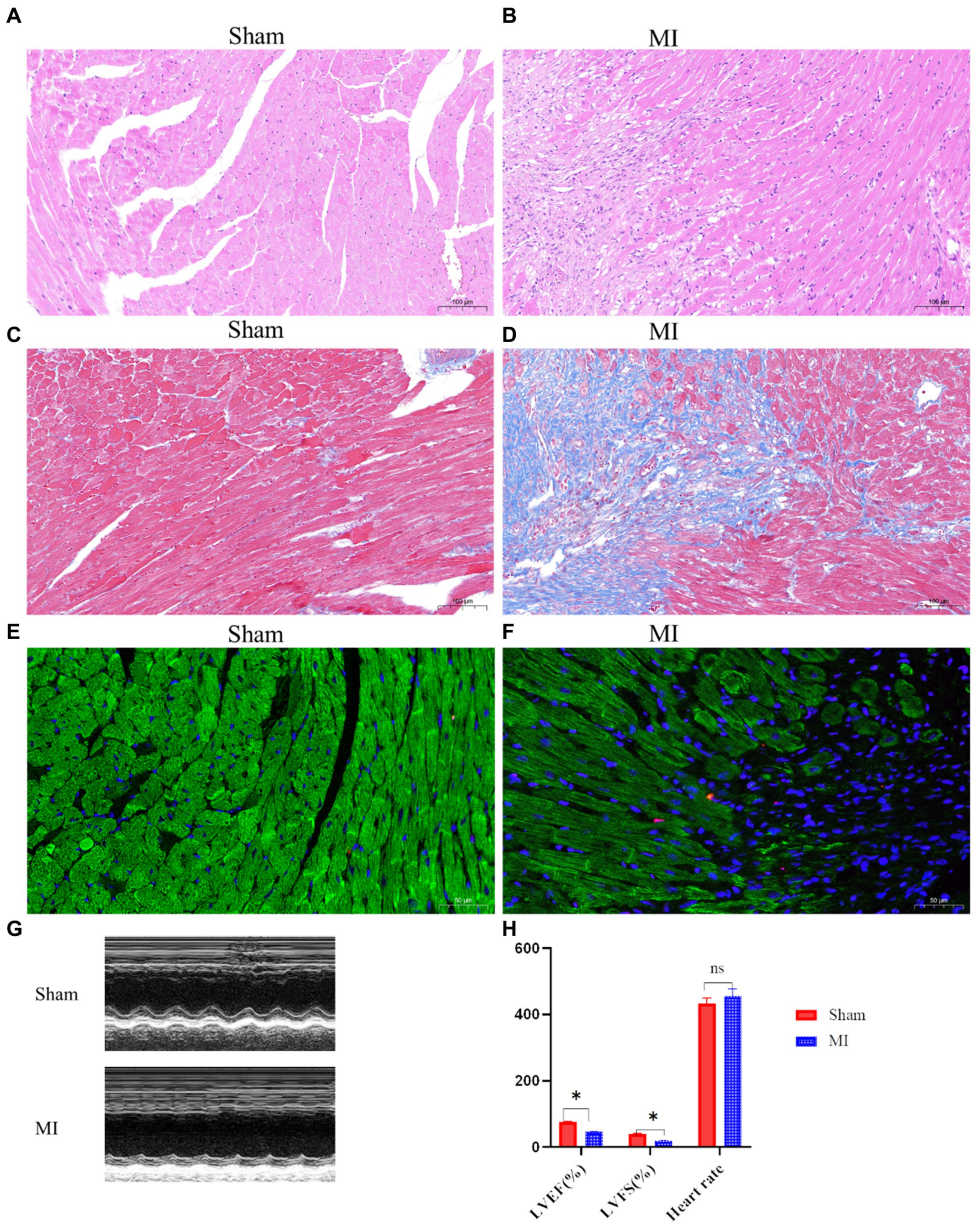
Cardiac function and histological changes in a mouse model of MI. **(A,B)** Hematoxylin and eosin (H&E) staining shows disrupted cardiac architecture and increased inflammatory cell infiltration in the MI group, whereas the sham group exhibits normal cardiac tissue morphology. **(C,D)** Masson’s trichrome staining reveals extensive fibrosis (blue areas) in the infarcted myocardium of the MI group, while minimal fibrosis is observed in the sham group. **(E,F)** Terminal deoxynucleotidyl transferase dUTP nick end labeling (TUNEL) staining indicates a higher number of apoptotic cells (dark nuclei) in the peri-infarct region of the MI group compared to the sham group. **(G,H)** Representative M-mode echocardiographic images demonstrate impaired left ventricular function in the MI group compared to the sham group.

H&E staining revealed disrupted cardiac architecture, increased inflammatory cell infiltration, and necrotic areas in the MI group (Figures 1A,B). Massons trichrome stain showed extensive fibrosis in the infarcted area of the MI group, with a significant increase in collagen deposition compared to the sham group (Figures 1C,D). TUNEL staining indicated a higher number of apoptotic cells in the peri-infarct region of the MI group, suggesting increased cell death following MI (Figures 1E,F). Echocardiography revealed that mice in the MI group have significantly reduced LVEF and LVFS compared to the sham group, indicating impaired left ventricular systolic function (Figures 1G,H).

### 3.2 Metabolomics analysis results

**Figure 2:**
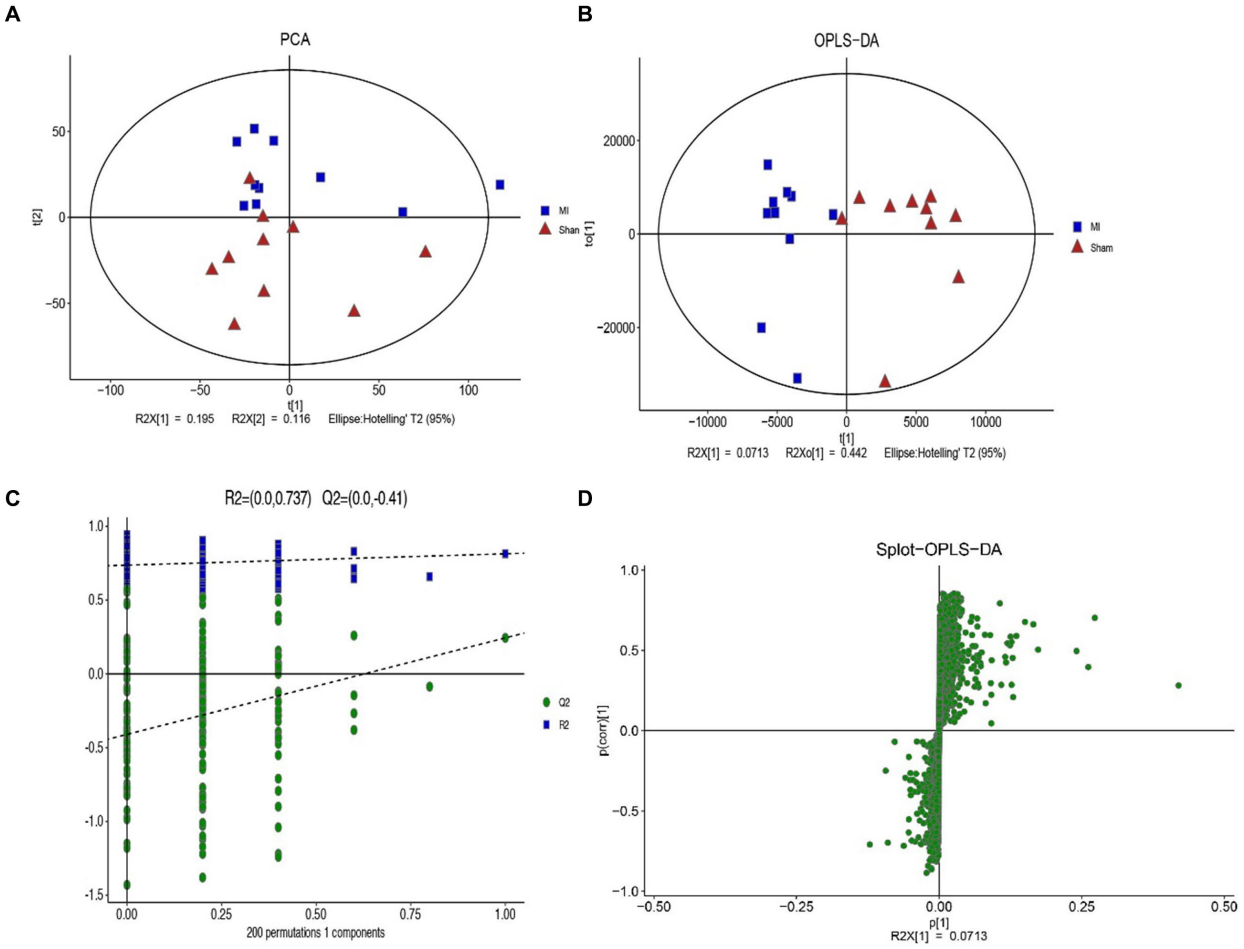
The difference of general serum metabolites with MI and sham group. **(A)** Principal component analysis (PCA) of general serum metabolites among two groups (R2X = 0.536). **(B)** OPLS-DA of serum metabolites R2X (*cum*) = 0.648, R2Y (*cum*) = 0.814. **(C)** The OPLS-DA permutation plot (R2 = 0.737, Q2 = −0.41). **(D)** S-plot of OPLS-DA modeling on MI-sham metabolic profiles.

**Figure 3:**
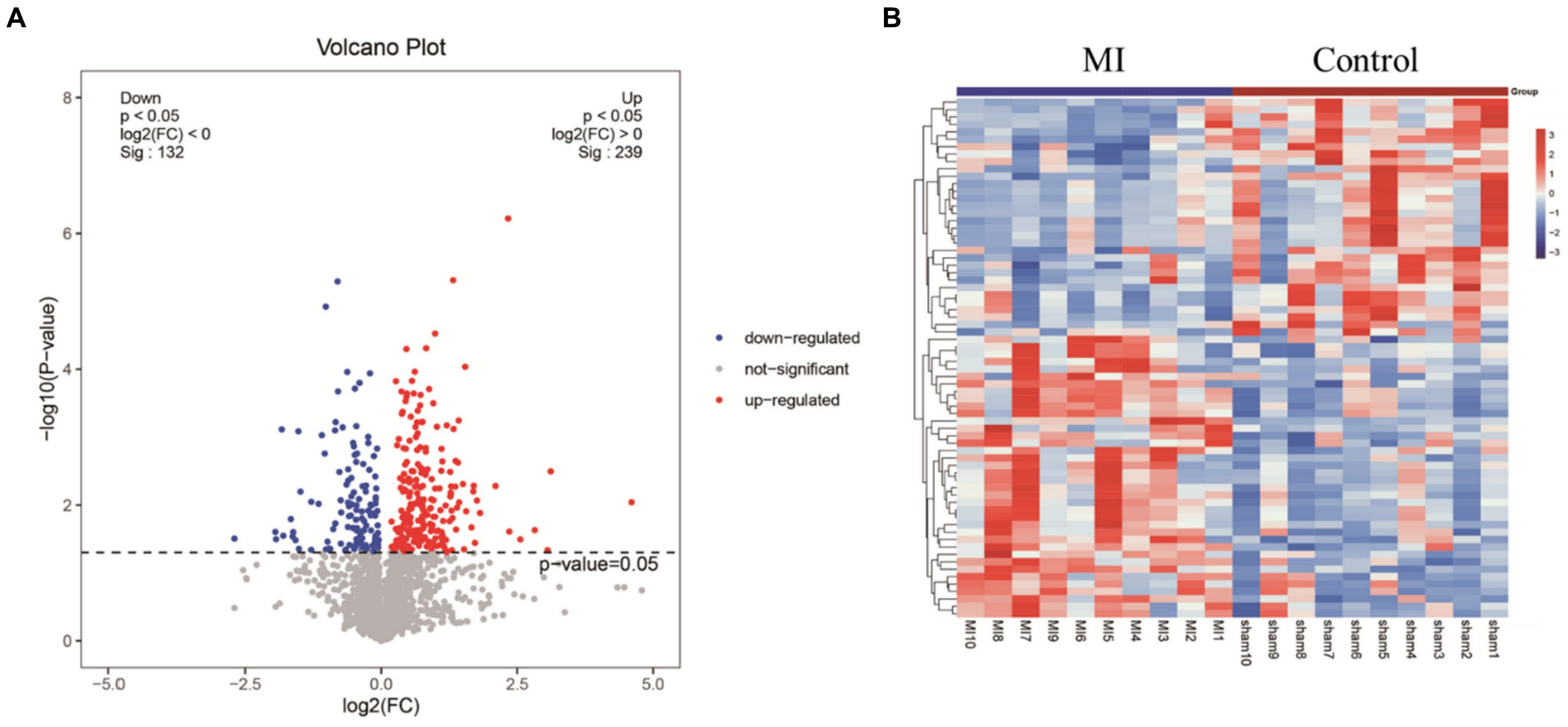
**(A)** Volcano plot highlights differentially expressed metabolite features between MI and sham group. The *x*-axis depicts fold change values between the two groups on a log scale, while the *y*-axis represents negative log transformed *p*-values from statistical significance tests. In this plot, up-regulated metabolites in MI locate to the right, whereas down-regulated species distribute to the left. **(B)** Hierarchical cluster heat map reveals distinct metabolite abundance patterns that discriminate between MI and sham group. Columns represent samples and rows denote metabolites. Red indicates metabolite upregulation, while blue signifies downregulation compared to the sham group.

**Figure 4:**
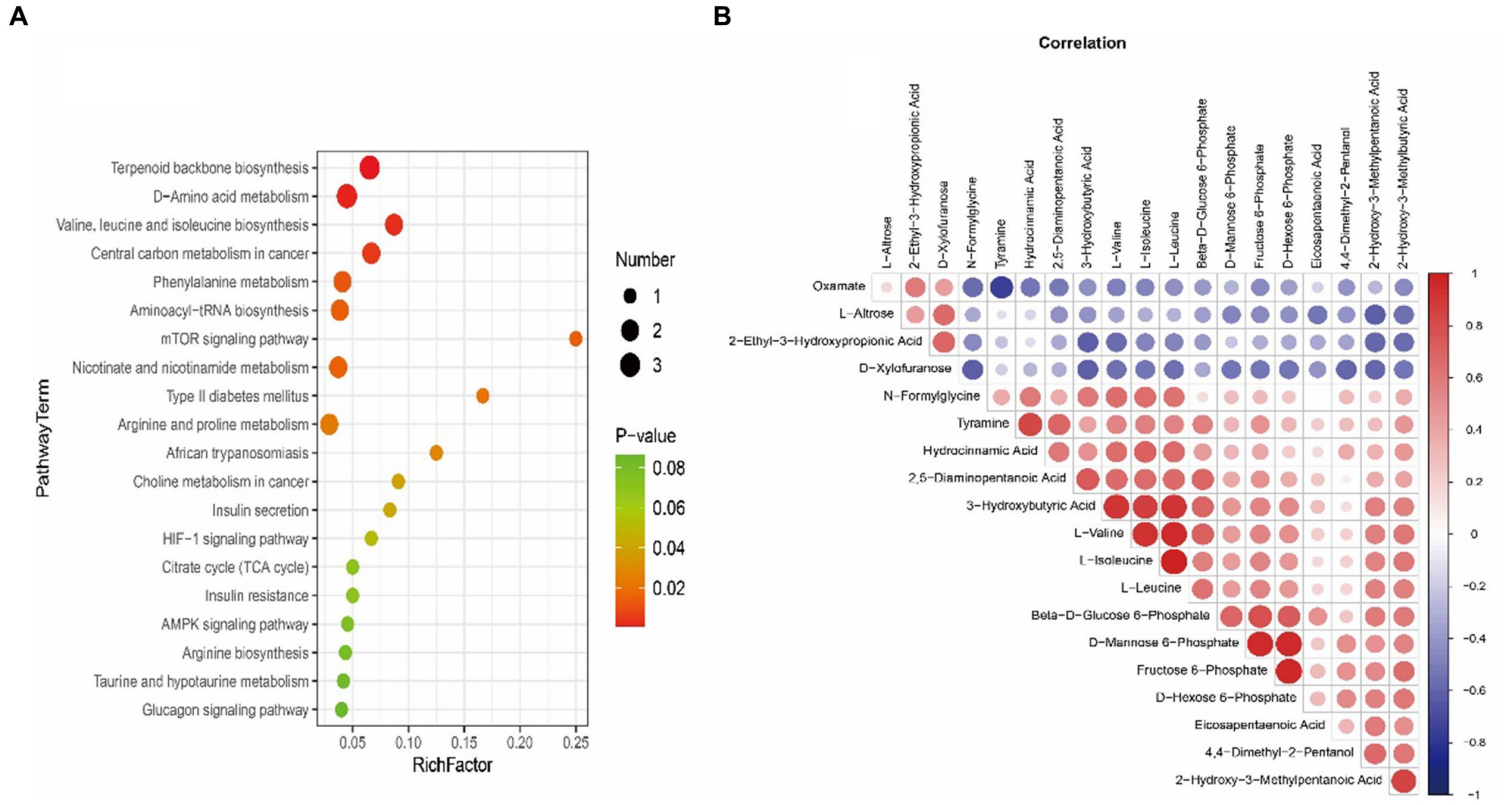
**(A)** Bubble plot exhibiting significantly enriched metabolic pathways associated with myocardial infarction (MI) identified through pathway enrichment analysis of differential metabolites. The *x*-axis denotes enrichment factors measuring pathway association strengths. Sizes of bubbles correlate with the number of enriched metabolites mapped to individual pathways. **(B)** Heatmap visualizing Spearman’s correlations between differential metabolites in myocardial infarction (MI) and sham groups. Positive correlations are shown in red and negative correlations in blue color gradients.

LC-MS technology was used to detect serum metabolomics profiles between both groups. After QC and standardization, approximately 3,000 features were obtained per sample. Unsupervised PCA clearly separated the two groups. OPLS-DA and random forest models verified good classification and prediction (Figure 2). Differential metabolites were sorted by VIP and validated by SAM, identifying 70 differences involving amino acid, lipid, carbohydrate pathways. Levels of Tetraazacyclododecanetetraacetic acid, MG (18:1(9Z)/0:0/0:0), 10-HDoHE, Acetyl-DL-Leucine, Isovalerylcarnitine, N-Acetyltryptophan, Allitridin, L-Leucine, etc. (Figure 3). Significantly increased in the MI group while PG (18:4/13:0), PA (12:0/20:4-2OH), PS (13:0/14:1), PA (a-13:0/20:5-OH(12)), PA (20:4-OH(11R)/14:1), PS (12:0/16:1), PC (16:0/5:0(CHO)), Trichloroethanol glucuronide, LysoPE (0:0/20:2(11Z, 14Z)), LysoPC (22:1(13Z)/0:0), 1-(9Z-Eicosenoyl)-sn-glycero-3-phosphocholine, D-Mannose etc. decreased (Table 1). Pathway analysis suggested microbial imbalance may have disturbed host D-Amino acid metabolism, Valine, leucine and isoleucine biosynthesis, Phenylalanine metabolism, Aminoacyl-tRNA biosynthesis and mTOR signaling pathway (Figure 4).

**Table 1:** Differential metabolites between myocardial infarction (MI) group and sham (Control) group.

*m*/*z*	Retention time (min)	Ion mode	Fragmentation Score	Metabolites	HMDB	VIP	*p*-value	log2(FC)
544.34	11.26	Pos	94.7	LysoPC (20:4(8Z, 11Z, 14Z, 17Z)/0:0)	HMDB0010396	23.72	2.32 × 10^−3^	−0.46
215.032	0.71	Neg	0	2-C-methyl-D-erythritol-4-phosphate	HMDB0304061	11.77	6.33 × 10^−3^	−0.28
1087.67	11.23	Pos	82.7	Bacterioruberin diglucoside		10.88	1.70 × 10^−2^	−0.32
132.102	1.23	Pos	71.6	L-Leucine	HMDB0000687	10.56	2.50 × 10^−3^	0.68
322.95	5.27	Neg	73.4	Trichloroethanol glucuronide	HMDB0042049	10.42	4.37 × 10^−2^	−0.96
550.387	12.61	Pos	95.9	1-(9Z-Eicosenoyl)-sn-glycero-3-phosphocholine	HMDB0243798	9.26	1.10 × 10^−4^	−0.62
166.086	1.98	Pos	52.2	Trans-cinnamic acid	HMDB0000930	7.81	3.49 × 10^−3^	0.42
217.029	0.71	Neg	0	Sulfanilamide	HMDB0014404	6.98	5.25 × 10^−3^	−0.29
552.403	13.64	Pos	87.8	(2-Acetyloxy-3-octadecoxypropyl) 2-(trimethylazaniumyl)ethyl phosphate	HMDB0242460	5.78	9.83 × 10^−3^	−0.55
118.087	0.77	Pos	79.8	Norvaline	HMDB0013716	5.39	2.32 × 10^−3^	0.47
518.325	10.91	Pos	90.1	LysoPC (18:3(6Z, 9Z, 12Z)/0:0)	HMDB0010387	5.01	2.49 × 10^−3^	−0.33
182.081	0.99	Pos	74	2-hydroxycinnamic acid	HMDB0002641	4.62	1.93 × 10^−2^	0.42
225.061	0.72	Neg	45.7	D-mannose	HMDB0000169	4.49	1.04 × 10^−2^	−0.59
205.097	4.09	Pos	41.1	Indoleacrylic acid	HMDB0000734	4.32	3.91 × 10^−2^	0.25
1111.67	11.24	Pos	8.84	Alpha-D-galactosyl undecaprenyl diphosphate		3.73	4.07 × 10^−2^	−0.48
147.113	0.62	Pos	98.7	L-Lysine	HMDB0000182	3.65	4.25 × 10^−4^	0.39
594.377	11.95	Pos	59.7	PC (16:0/5:0(CHO))		3.34	4.51 × 10^−2^	−1.02
528.309	11.38	Pos	87.8	LysoPE (22:5(4Z, 7Z, 10Z, 13Z, 16Z)/0:0)	HMDB0011524	2.95	9.72 × 10^−3^	0.54
664.419	13.26	Pos	0	PS (13:0/14:1(9Z))		2.91	3.18 × 10^−2^	−1.93
316.948	0.63	Neg	1.48	Beta-D-fructose 2,6-bisphosphate	HMDB0304539	2.47	7.69 × 10^−3^	−0.10
167.09	1.98	Pos	1.27	3-(4-methyl-3-pentenyl)thiophene	HMDB0038183	2.43	4.42 × 10^−3^	0.41
592.363	11.97	Neg	98.7	LysoPC (20:2(11Z, 14Z)/0:0)	HMDB0010392	2.29	6.90 × 10^−4^	−0.46
130.086	0.62	Pos	96.9	D-pipecolic acid	HMDB0005960	2.12	4.52 × 10^−4^	0.38
578.418	13.82	Pos	0	LysoPC (22:1(13Z)/0:0)	HMDB0010399	2.10	4.99 × 10^−3^	−0.64
383.116	0.69	Pos	0	Invert sugar	HMDB0303239	2.00	4.36 × 10^−3^	−0.60
158.978	0.59	Neg	0	Allitridin	HMDB0031154	1.99	1.41 × 10^−3^	0.72
338.087	4.92	Pos	81.9	2,8-dihydroxyquinoline-beta-D-glucuronide	HMDB0011658	1.98	2.28 × 10^−3^	1.12
178.05	5.00	Neg	75.2	4-acetamidobenzoic acid	HMDB0246328	1.93	1.16 × 10^−2^	0.51
662.307	5.23	Pos	8.01	PHODiA-PE		1.77	5.01 × 10^−4^	0.55
140.068	0.70	pos	23.3	N,N-dimethyl-beta-alanine	HMDB0304421	1.73	1.18 × 10^−2^	−0.46
686.439	13.09	Pos	0	PA (a-13:0/20:5(5Z, 8Z, 10E, 14Z, 17Z)-OH(12))	HMDB0266859	1.67	1.61 × 10^−2^	−1.66
690.434	12.21	Pos	0	PA (12:0/20:4(6E, 8Z, 11Z, 13E)-2OH(5S, 15S))	HMDB0262818	1.65	2.49 × 10^−2^	−1.95
718.465	13.19	Pos	0	PG (18:4(6Z, 9Z, 12Z, 15Z)/13:0)		1.64	3.12 × 10^−2^	−2.70
357.3	13.57	Pos	82.9	MG (18:1(9Z)/0:0/0:0)	HMDB0011567	1.63	5.70 × 10^−3^	1.07
711.318	5.22	Neg	0	S-adenosyl-L-methioninamine	HMDB0304476	1.60	1.84 × 10^−2^	0.54
605.322	11.28	Neg	0	Tribenzylamine n-oxide		1.57	1.57 × 10^−2^	−0.35
136.076	0.99	Pos	12.9	Benzofuran	HMDB0032929	1.56	1.86 × 10^−2^	0.43
136.076	0.81	Pos	5.97	2,4,6-octatriyn-1-ol	HMDB0030968	1.54	1.35 × 10^−2^	0.42
596.394	13.66	Neg	85.3	LysoPC (20:0/0:0)	HMDB0010390	1.53	3.87 × 10^−2^	−0.58
123.056	0.91	Pos	78.4	Niacinamide	HMDB0001406	1.51	3.90 × 10^−2^	−0.28
206.1	4.09	Pos	0	METHYL (-)-SHIKIMATE; methyl 3,4,5-trihydroxy-1-cyclohexene-1-carboxylate		1.51	4.45 × 10^−2^	0.25
327.051	0.63	Pos	0	Lamivudine-monophosphate	HMDB0060641	1.50	1.07 × 10^−3^	0.32
276.018	1.24	Neg	60.6	2,5-dioxopyrrolidin-1-yl 2-(acetylthio)acetate	HMDB0247382	1.47	9.64 × 10^−3^	0.61
620.392	12.15	Pos	0	Dolichyl beta-D-glucosyl phosphate		1.46	2.50 × 10^−2^	−1.62
660.423	12.55	Pos	0	PS (12:0/16:1(9Z))		1.46	4.40 × 10^−2^	−1.51
621.305	11.75	Neg	0	OHOHA-PA		1.46	2.19 × 10^−2^	−0.44
117.054	3.40	Neg	0	1,2-Diacylglycerol-LD-PE-pool	HMDB0062269	1.45	1.50 × 10^−4^	0.27
133.097	0.62	Pos	93.5	Ornithine	HMDB0000214	1.44	9.93 × 10^−4^	0.64
646.408	12.33	Pos	0	PA (10:0/20:3(5Z, 8Z, 14Z)-O(11S, 12R))	HMDB0262641	1.44	4.57 × 10^−2^	−1.29
528.308	11.56	Pos	93.8	LysoPE (22:5(7Z, 10Z, 13Z, 16Z, 19Z)/0:0)	HMDB0011525	1.42	4.06 × 10^−2^	0.42
700.455	13.19	Pos	0	PA (20:4(5E, 8Z, 12Z, 14Z)-OH(11R)/14:1(9Z))	HMDB0263079	1.42	3.30 × 10^−2^	−1.57
244.079	0.69	Pos	0	Pyruvic acid	HMDB0000243	1.39	1.83 × 10^−3^	−0.49
176.103	0.70	Pos	0	N-nitrosobis (2-oxopropyl)amine	HMDB0255200	1.39	4.03 × 10^−3^	0.35
827.75	7.96	Pos	0	TG (15:0/17:1(9Z)/19:1(9Z))[iso6]		1.34	1.09 × 10^−4^	0.62
530.324	11.77	Pos	0	LysoPE (22:4(7Z, 10Z, 13Z, 16Z)/0:0)	HMDB0011523	1.31	7.52 × 10^−3^	0.51
180.066	5.00	Pos	67.3	Hippuric acid	HMDB0000714	1.31	1.39 × 10^−2^	0.48
246.17	4.98	Pos	88.1	Isovalerylcarnitine	HMDB0000688	1.20	1.62 × 10^−2^	0.75
244.998	1.18	Pos	0	(2E)-4-hydroxy-3-methylbut-2-en-1-yl trihydrogen diphosphate		1.18	6.33 × 10^−3^	0.40
247.108	5.89	Pos	99.4	N-acetyltryptophan	HMDB0013713	1.17	3.36 × 10^−3^	0.74
343.228	11.72	Neg	73.6	10-HDoHE	HMDB0060037	1.17	3.90 × 10^−2^	0.98
405.198	3.73	Pos	0	Tetraazacyclododecanetetraacetic acid	HMDB0257787	1.16	4.89 × 10^−6^	1.32
172.097	5.52	Neg	75.6	Acetyl-DL-leucine		1.16	2.99 × 10^−3^	0.85
614.346	6.14	Pos	1.4	OKOOA-PE		1.13	4.64 × 10^−3^	0.52
579.306	10.92	Neg	0	Hordatine B	HMDB0030459	1.13	5.28 × 10^−3^	−0.34
425.081	0.71	Neg	76.8	Cysteineglutathione disulfide	HMDB0000656	1.13	4.27 × 10^−2^	0.50
269.055	0.74	Neg	0	2-Cyano-3-oxo-n-[4-(trifluoromethyl)phenyl]butanamide	HMDB0257847	1.10	8.21 × 10^−4^	−1.52
506.325	11.89	Pos	0	LysoPE (0:0/20:2(11Z, 14Z))	HMDB0011483	1.08	7.18 × 10^−4^	−0.71
229.155	0.97	Pos	22.1	Pro-Ile	HMDB0304810	1.08	3.74 × 10^−2^	0.42
395.039	0.64	Pos	0	Caffeic acid 3-O-glucuronide	HMDB0041705	1.04	1.32 × 10^−3^	0.29
209.092	1.92	Pos	97.1	L-kynurenine	HMDB0000684	1.02	2.28 × 10^−4^	0.47

### 3.3 Microbiological analysis results

**Figure 5:**
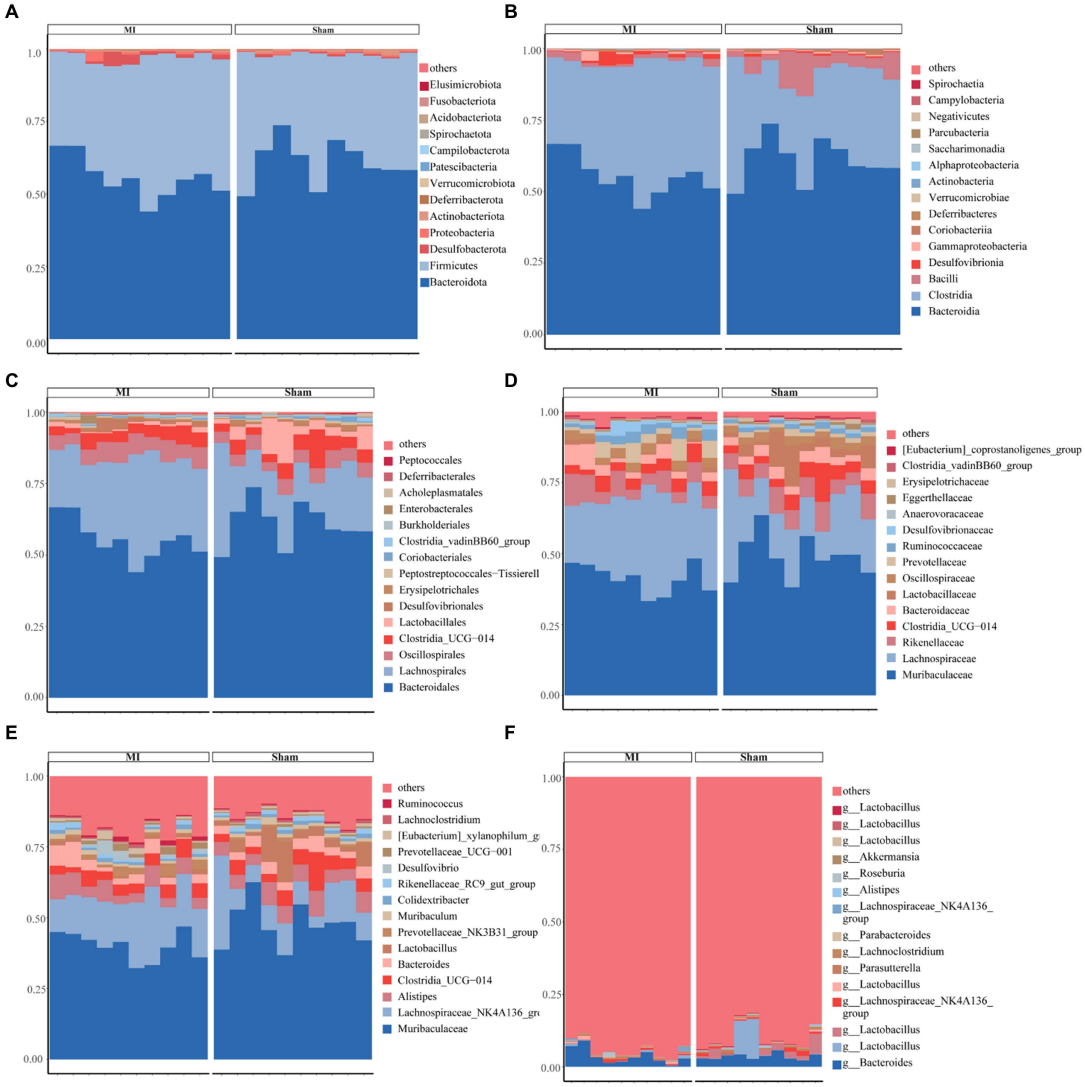
Bar plots exhibiting differential abundance of gut microbiota between MI and sham. **(A)** Phylum level: Bacteroidota and Firmicutes as dominant phyla. **(B)** Class level: Bacteroidia and Clostridia as dominant gut microbes. **(C)** Order level: Bacteroidales and Lachnospirales as dominant gut microbes in MI and sham controls. **(D)** Family level. **(E)** Genus level. **(F)** Species level.

This project involved a total of 20 samples. In the differential statistical analysis of the project samples, using the *t*-test algorithm, we identified 125 differential ASVs, 24 differential genera, and 1 differential phylum. Meanwhile, employing the Wilcoxon algorithm resulted in 165 differential ASVs, 25 differential genera, and 2 differential phyla. The microbial composition of the thrombi varied widely across individuals. *Bacteroidota (p)* and *Firmicutes (p)* were the most abundant phyla among the detected microbes (Figure 5). We found 244 species, 151 families, 48 orders, 20 classes, and 14 phyla. Figure 5 displays the general landscape of the microbial composition of all samples at the genus level. Furthermore, taxonomic assignments at the other levels (phylum, class, order, family, and species) are also shown in Figure 5.

**Figure 6:**
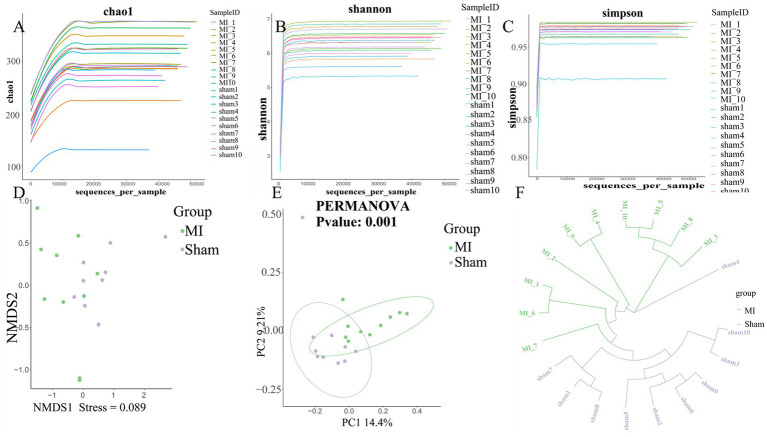
Comprehensive diversity analysis of microbial communities. **(A)** Alpha diversity, including **(A)** Chao; **(B)** Shannon index and **(C)** Simpson index; **(D)** Non-metric Multidimensional Scaling (NMDS) analysis; **(E)** Beta diversity analysis using Principal Coordinates Analysis (PCoA). **(F)** UPGMA Hierarchical Clustering Dendrogram of Samples.

First, microbial diversity was measured. The alpha-diversity of the stool microbiome, defined as the number of species present within each sample, did not differ significantly between groups (Figure 6). We assessed the relationships among bacterial communities from their beta-diversity to generate PCoA plots. OTUs of the thrombus microbiome relative to those of the gut and oral microbiomes displayed different clustering between groups (Figure 6), suggesting phylogenetic closeness between microbial communities within each group.

**Figure 7:**
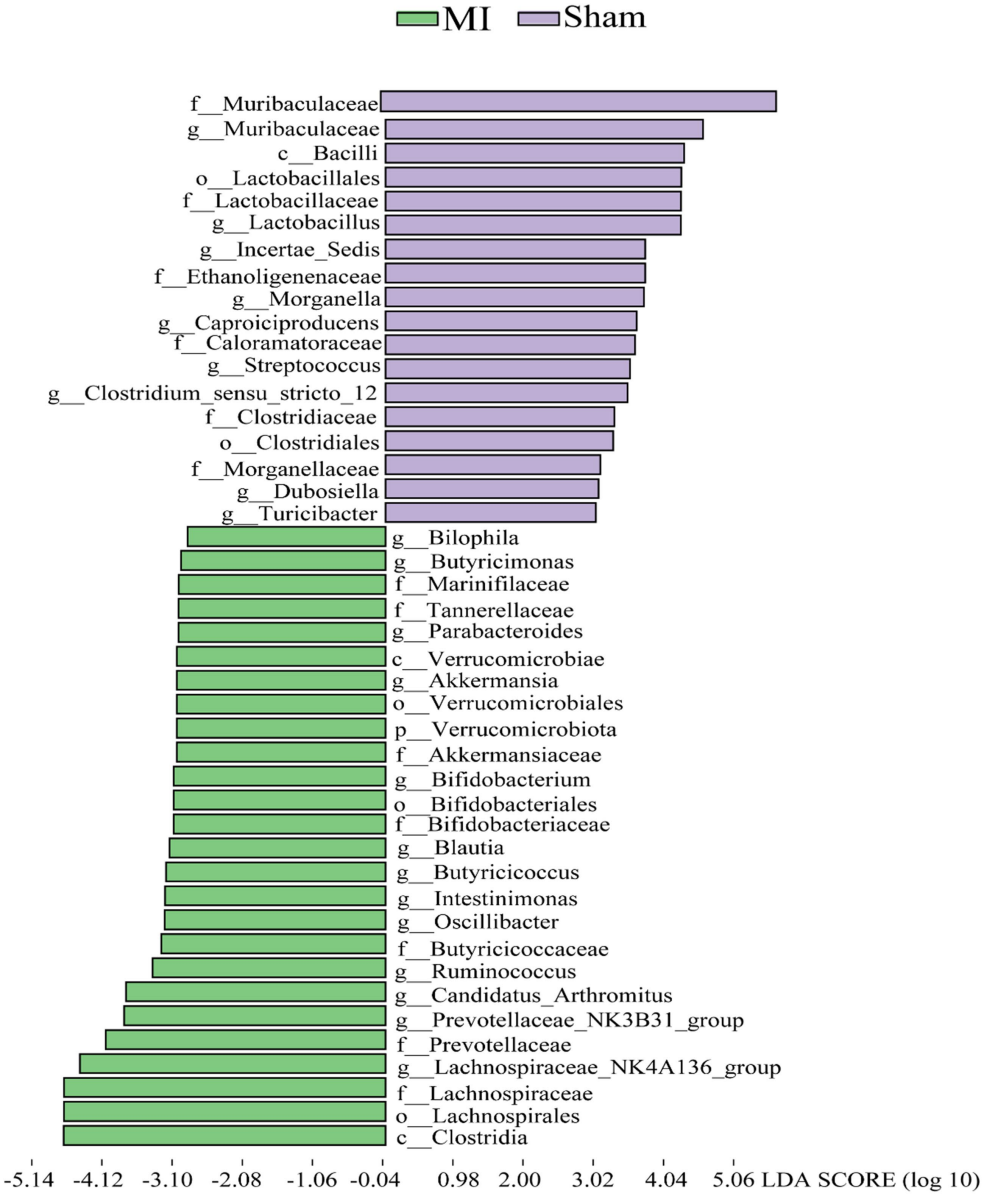
Histogram of the LDA scores for differentially abundant bacterial taxa in the stool sample between the MI group and sham groups. Green and Purple colors represent bacterial taxa that were significantly overrepresented in the MI group and sham groups, respectively.

**Figure 8:**
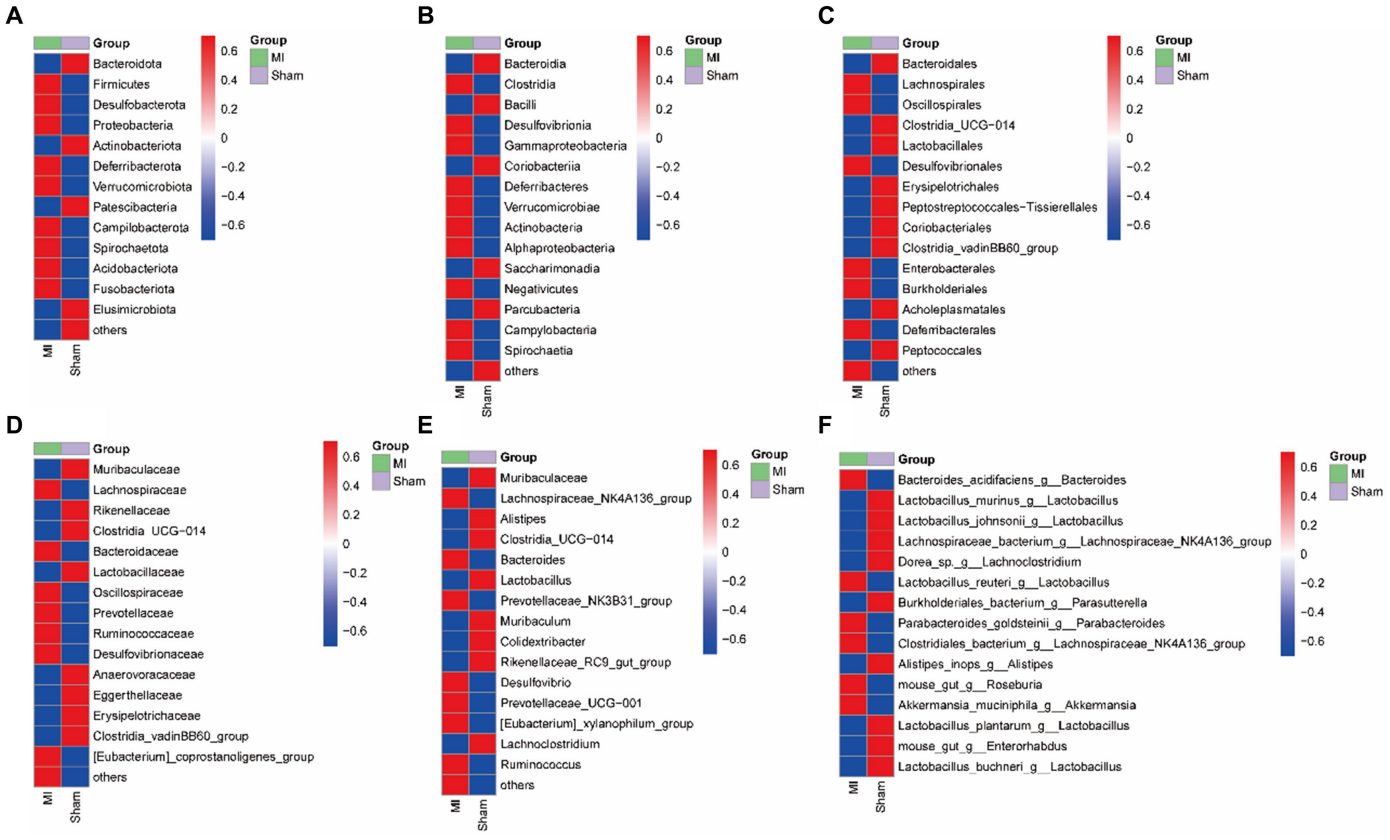
Heatmap of the selected most differentially abundant features at the **(A)** phylum; **(B)** class; **(C)** order; **(D)** family; **(E)** genus; **(F)** species levels. Blue represents lower abundance and red represents the highest abundance.

LEfSe screened 81 clades from the gut microbiome. The MI group displayed dysbiosis of the gut microbiota. The prevalence of *Verrucomicrobiota (p)* was significantly higher in the MI group than in the sham group (LDA = 3.03, *p* = 0.049), *Blautia(g), Bilophila(g), Butyricicoccus(g), Oscillibacter(g), Intestinimonas(g), Bifidobacterium(g), Ruminococcus(g), Butyricimonas* and *Parabacteroides(g)* were significantly higher in the MI group than in the sham group, while that of *Bacilli (c), Clostridiales (o), Lactobacillaceae (f), Lactobacillus (g), Morganella, Turicibacter, Incertae_Sedis, Dubosiella, Muribaculaceae, Caproiciproducens* and *Streptococcus* were significantly lower in MI group. Short-chain fatty acid (SCFA)-producing bacteria displayed a higer abundance (*Prevotella (g); Lachnospiraceae (f)*) in MI group (Figure 7). The heatmap of the selected most differentially abundant features at the (A) phylum; (B) class; (C) order; (D) family; (E) genus; (F) species levels (Figure 8).

### 3.4 Different metabolic pathways of microbial communities between MI and sham group

**Figure 9:**
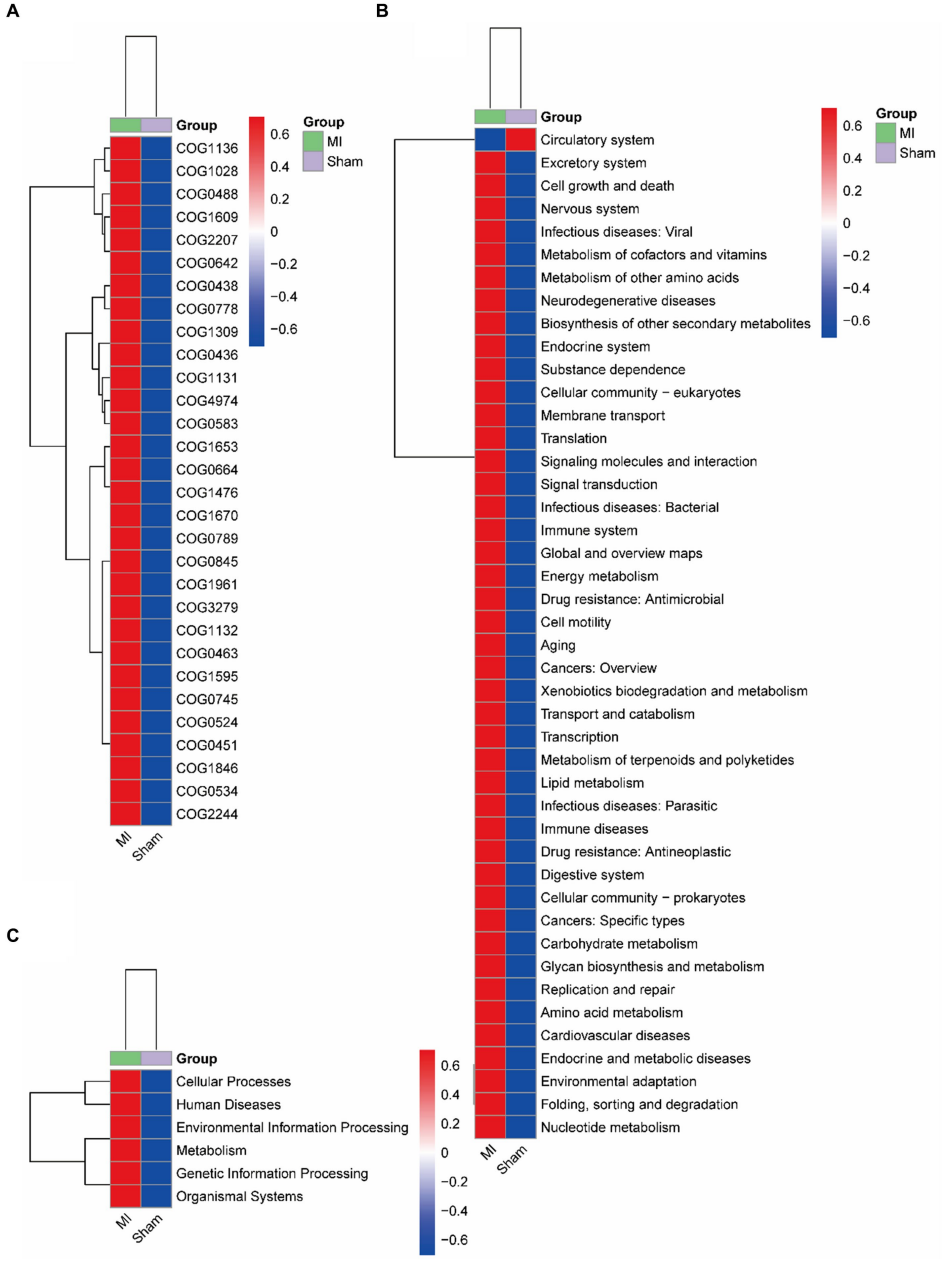
Metabolic implication. Different metabolic pathways of microbial communities between the: a gut microbiome and b oral microbiome groups using the PICRUSt algorithm.

Bacterial gene functions were predicted via 16S rRNA gene-based microbial compositions using the PICRUSt algorithm to make inferences from KEGG annotated databases. The MI and sham group exhibited significant differences among the 41 KEGG pathways (Figure 9). The MI microbiome displayed differences in Cellular Processes, Environmental Information Processing, Genetic Information Processing, Human Diseases, Metabolism and Organismal Systems in level 1. The MI microbiome displayed differences in amino acid metabolism, Carbohydrate metabolism, Cardiovascular diseases, Circulatory system, Cell motility and biosynthesis of other secondary metabolites in level 2.

### 3.5 Multi-omics association analysis results

**Figure 10:**
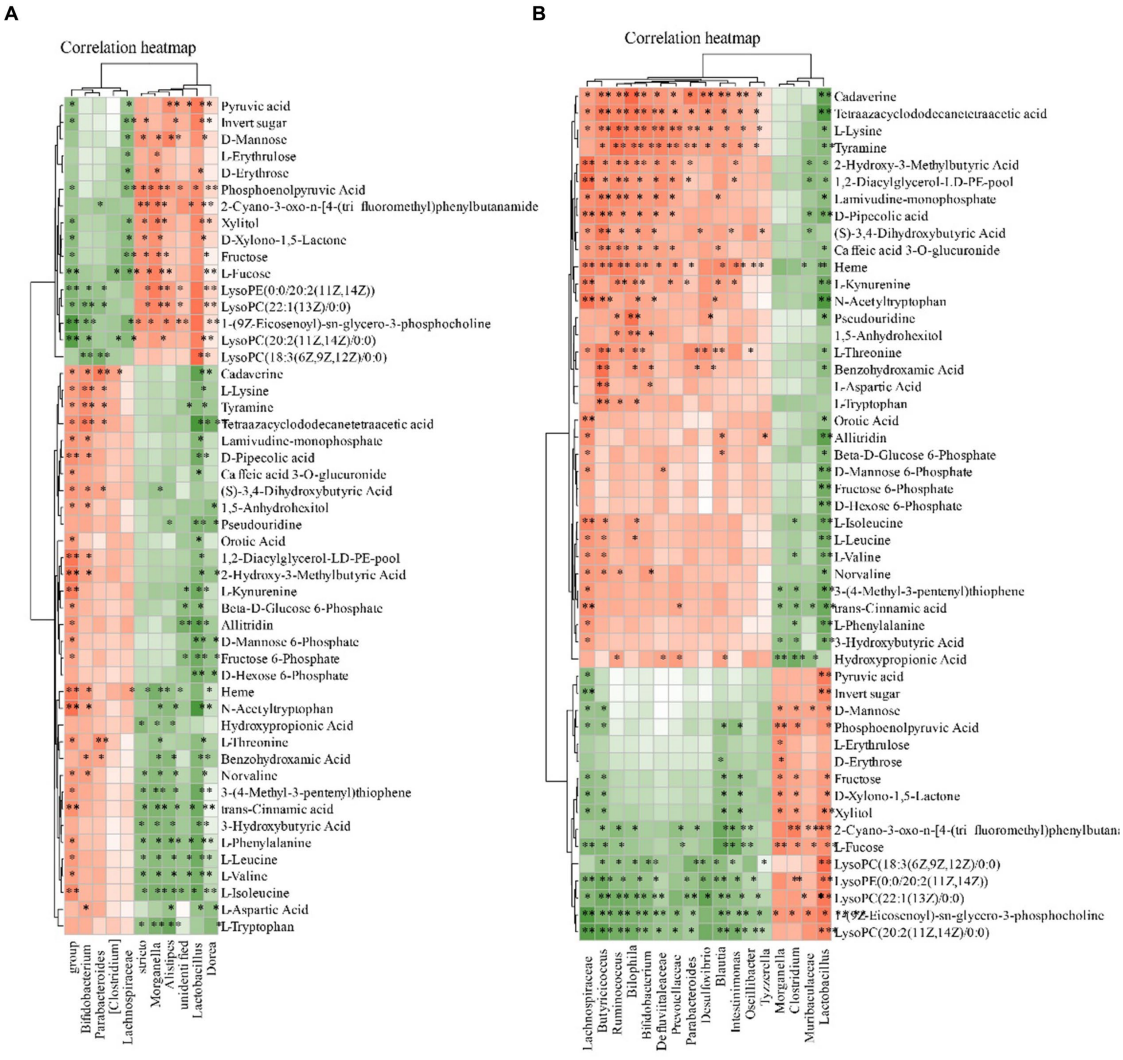
Relationship between the gut microbiota and serum metabolites. **(A)** Heatmap analysis of microbial-associated metabolite profiles at species level. **(B)** Heatmap analysis of microbial-associated metabolite profiles at genus level. The color gradient represents the strength and direction of associations: red indicates positive correlation, blue indicates negative correlation. Columns represent individual microbial taxa, and rows represent specific metabolites identified.

We subsequently correlated the gut microbiota with serum metabolites to further explore the characteristics of microbiota in MI. The gut microbiota was further correlated with the blood metabolites. Eight blood metabolites, L-Leucine, L-Valine, L-Isoleucine, L-Lysine, L-Aspartic Acid, Ornithine Pyruvic acid and they were positive related with fecal contents of *Ruminococcus, Intestinimonas, Lactobacillus, Parabacteroides, Bifidobacterium, Desulfovibrio, Blautia* (*p* < 0.05). These metabolites were negative to *Clostridium and Lactobacillus* (Figure 10).

## 4 Discussion

Myocardial infarction (MI), universally recognized as a heart attack, remains at the forefront of global health challenges, contributing significantly to the morbidity and mortality rates worldwide, with an estimated annual incidence nearing 6 million cases (Amini et al., 2021). The pathogenesis of MI is characterized by a complex interplay involving lipid accumulation, inflammation, platelet activation, oxidative stress, and electrolyte imbalance, which collectively precipitate the acute rupture of coronary plaques and subsequent thrombosis (Smit et al., 2020).

Despite considerable advancements in medical interventions for MI, individuals recovering from such a cardiac event continue to face a heightened risk of recurrent ischemia and related comorbidities, which notably impair their quality of life. Emerging omics technologies promise a paradigm shift towards personalized healthcare, aiming to transcend the traditional one-size-fits-all approach. However, the underlying mechanisms of MI pathogenesis require further elucidation to inform the development of novel therapeutic interventions (Zhan et al., 2023). Among the novel discoveries in this domain is the “gut-heart axis,” a testament to the burgeoning recognition of the intestinal microenvironment’s influence on cardiovascular health (Moldovan et al., 2022). The gut microbiome, through its metabolic outputs, has been implicated in MI pathogenesis, affecting endothelial function, atherosclerosis development, thrombosis, and myocardial damage (Xu et al., 2023). Preliminary trials indicate that interventions aimed at restoring eubiosis-whether through nutritional or microbial means—have shown promise in mitigating MI effects. The identification of gut microbiome signatures specific to MI could herald the advent of novel diagnostic biomarkers and therapeutic targets (Dekkers et al., 2022). In conclusion, understanding the decoding mechanistic microbiome-metabolome interplay that governs MI opathology holds the potential to transform future prediction, prevention and precision management of this global threat. Therefore, multi-modal strategies targeting holistic gut-heart axis represent an innovative frontier to attenuate MI severity (Chen et al., 2023).

The serum metabolites identified in this study included amino acids like proline, branched-chain amino acids (Leucine, Isoleucine and Valine); lipids such as monoacylglycerols (MG), triacylglycerols (TG); neurotransmitter precursors for example tryptophan and kynurenine. Meanwhile, microbes belonging to common gut commensals and short chain fatty acids (SCFA) producers were detected, including *Bifidobacterium, Lactobacillus, Ruminococcus*.

Branched-chain amino acids (BCAAs), including leucine, isoleucine, and valine, are essential amino acids that undergo degradation through a shared metabolic pathway. The breakdown of BCAAs produces branched-chain α-keto acids (BCKAs), including α-ketoisocaproate (KIC), α-keto-β-methylvalerate (KMV), and α-ketoisovalerate (KIV). Defects in BCAA metabolism and elevated levels of BCKAs or BCAAs have been reported in pathologically stressed mouse hearts and human cardiomyopathy (Walejko et al., 2021; McGarrah and White, 2023). In cardiac muscle cells, they impede the activity of mitochondrial Complex I and induce the production of superoxides (McGarrah and White, 2023). Deficiencies in BCAA metabolism significantly contribute to heart failure and myocardial remodeling following chronic pressure overload or myocardial infarction. Mechanistic studies have revealed that BCKAs protect mitochondria and energy production, aiding in cell survival (Karwi and Lopaschuk, 2023). Importantly, BCKAs demonstrate protective effects in both cultured cells and intact hearts, regardless of whether administered as a pre-treatment or post-treatment (Dong et al., 2016). Previous studies have shown that BCKAs alleviate acute ischemic/reperfusion (I/R) injury in the heart, identifying a novel anti-necrotic function of BCAA metabolites and suggesting potential applications of BCKA-like compounds in the treatment of ischemic diseases (Dong et al., 2016).

This study reports an increased abundance of *Verrucomicrobiota (p), Blautia(g), Bilophila(g), Butyricicoccus(g), Oscillibacter(g), Intestinimonas(g), Bifidobacterium(g), Ruminococcus(g), Butyricimonas* and *Parabacteroides(g)*. These diverse microbial ecosystems undertaking key physiological roles like nutrient digestion, vitamin synthesis, and immune modulation. The microbes, such as short chain fatty acid (SCFA)-producing *Blautia, Butyricicoccus* and *Bifidobacterium*; mucin-degraders like *Oscillibacter*; carbohydrate fermenting *Ruminococcus* and *Intestinimonas*. The major fermentation products of these bacteria, including SCFAs like butyrate and acetate, can be absorbed to provide energy, enhance gut barrier integrity, and regulate immunity. However, under disease states like myocardial infarction, the gut microbiota equilibrium gets disrupted, allowing expansion of opportunistic pathogens like *Bilophila*. This coupled with reduced beneficial symbionts may damage intestinal lining, increase exposure to microbial antigens and metabolites, ultimately overactivating inflammatory and oxidative pathways that accelerate cardiovascular damages. Therefore, characterization of myocardial infarction-associated gut ecosystem changes in a holistic manner is crucial. This allows better understanding of the microbial mediators and mechanisms contributing to cardiovascular pathogenesis. Targeted restoration of key commensals deficient in myocardial infarction patients through probiotics or prebiotics may mitigate gut dysfunction, dampen systemic inflammation, and improve disease outcomes.

The interconnections between gut microbiota, cardiovascular diseases, and renal function highlight the significant impact of gut-derived metabolites on general health. Gut-derived uremic toxins, such as indoxyl sulfate, p-cresyl sulfate, kynurenine, and kynurenic acid, have been identified as harmful metabolites that can lead to cardiovascular diseases in individuals with chronic kidney disease. These metabolites are produced by the gut microbiota and are problematic when kidney function is compromised, leading to their accumulation (El Chamieh et al., 2022). Prebiotics, which foster beneficial gut bacteria, can reduce the production of harmful uremic toxins like indoxyl sulfate and p-cresyl sulfate. These toxins are produced by pathogenic gut bacteria and have significant implications for kidney and cardiovascular health (Falconi et al., 2021). A healthy gut microbiota, supported by prebiotics, plays a vital role in managing these toxins, thereby indirectly supporting both kidney and cardiovascular health (Mitrea et al., 2023).

Our research conducted utilizing a multi-omics approach is comprehensive; however, it is constrained by a limited cohort size that may impede the generalizability of the results. Subsequent investigations could improve upon this limitation by including larger and more diverse study populations, potentially progressing to human clinical trials. Despite the aforementioned limitations, our study makes significant contributions to the field of microbiota research, specifically in elucidating the microbiota-metabolite-myocardium axis. Through the novel application of integrative multi-omics techniques, we conducted a thorough analysis of intricate interactions, yielding fresh perspectives on the impact of post-infarction gut microbiota alterations on myocardial health. This study expands upon existing knowledge and proposes potential clinical implications, including the identification of new biomarkers and therapeutic targets, which may enhance patient care in the context of cardiovascular diseases.

## 5 Conclusion

This study conducted a comprehensive analysis of the gut microbiomes and serum metabolites in myocardial infarction (MI) and sham groups, revealing significant differences in the composition and function of the microbiomes between the two groups. The findings suggest that microbial dysbiosis might be a contributing mechanism to acute myocardial infarction. The research also discovered microbial communities within human coronary thrombi, which correlated with those in the gut and oral microbiomes. These results pave the way for future research to further understand the clinical implications and to develop new therapeutic approaches. In conclusion, this study provides preliminary evidence that gut dysbiosis is involved in the pathogenesis of myocardial infarction via microbiota-metabolite axes, laying a foundation for new treatment strategies.

## Data availability statement

The 16S rDNA has been deposited to NCBI with the accession number PRJNA1102168. The raw date of metabolomics has been deposited to MetaboLights with the accession number MTBLS9620.

## Ethics statement

The animal study was approved by the Ethics Committee of Shibei Hospital. The study was conducted in accordance with the local legislation and institutional requirements.

## Author contributions

ZG: Writing – original draft. YZ: Project administration, Writing – original draft. LZ: Investigation, Software, Writing – review & editing. PX: Data curation, Validation, Writing – original draft. NG: Validation, Visualization, Writing – review & editing. JL: Investigation, Methodology, Writing – original draft. XY: Supervision, Visualization, Writing – original draft. HC: Funding acquisition, Project administration, Resources, Writing – original draft, Writing – review & editing.

## Footnotes

^1^ https://metorigin.met-bioinformatics.cn/home/
